# Competency in responding to infectious disease outbreaks among nurses in primary healthcare institutions: a quantitative, cross-sectional multicentre study

**DOI:** 10.3389/fpubh.2024.1406400

**Published:** 2024-07-22

**Authors:** Wei Zhu, Jizhen Zhang, Liyao Yang, Jiping Li, Hongxia Guo

**Affiliations:** ^1^Department of Neurosurgery, West China Hospital, Sichuan University, Chengdu, China; ^2^West China School of Nursing, Sichuan University, Chengdu, China; ^3^Department of Nursing, West China Hospital, Sichuan University, Chengdu, China

**Keywords:** competency, infectious disease, primary healthcare, nurse practitioner, cross-sectional studies

## Abstract

**Background:**

Nurses’ competencies are crucial for infectious disease prevention and control. We aimed to investigate competencies in responding to infectious disease outbreaks of nurses in primary healthcare institutions and identify their training needs.

**Methods:**

A cross-sectional study was conducted from June to September 2022, recruiting nurses from primary healthcare institutions across Sichuan Province. Their competencies and training needs were assessed using a modified Emergency Response Competency Scale for Infectious Diseases. Additionally, their sociodemographic characteristics and experience in infectious disease outbreak trainings were collected. Univariate analyses were used to compare competencies and training needs by participant characteristics. Multiple linear regression was conducted to identify determinants of their competencies.

**Results:**

A total of 1,439 nurses from 44 primary healthcare institutions participated in this study. The overall competency and training needs had a median of 3.6 (IQR [3.1, 4.0]) and 4.0 (IQR [3.9, 4.7]), respectively. Age (*β* = −0.074, *p* = 0.005), experience in higher authority hospitals (*β* = 0.057, *p* = 0.035), infectious disease outbreak trainings attended within the last 5 years (*β* = 0.212, *p* < 0.001), and regions where the institutions located were determinants of the competencies.

**Conclusion:**

The competencies in responding to infectious disease outbreaks among nurses in primary healthcare institutions were at a moderate level, influenced by varied factors.

## 1 Introduction

The prevalence of infectious diseases with pandemic potential has been increasing in recent years, which may develop into a global health threat due to the rapid spread of these diseases through cross-border travel and workforce migration (1). Despite significant efforts and considerable progress made in the prevention and control of infectious diseases, they continue to pose significant threats to public health systems and economies globally (1, 2). According to a report from the China Disease Prevention and Control Centre, the incidence of infectious diseases in China was 442.16 per 100,000 individuals, with a mortality rate of 1.57 per 100,000 in 2021 (3, 4). To effectively address the growing threats posed by infectious disease outbreaks to public health systems and economies, it is important to enhance the surveillance and response capacities of not only China but also public health systems worldwide (5).

As the cornerstone of public health systems, primary healthcare institutions usually bear the responsibility of early screening symptomatic patients, providing proper control of infectious diseases, ensuring safe transfers, and minimizing the requirement of hospital services as much as possible (6, 7). The capacities of primary healthcare institutions for response to infectious disease outbreaks become even more important when decentralized infectious disease prevention and treatment strategies are implemented to reduce access disparities in medical services (8). Actually, the capacities of public health systems for response to infectious disease outbreaks do not solely rely on contingency plans or equipment held by institutions. It also encompasses the preparedness of frontline healthcare providers, including their preparedness of skills, knowledge, and attitudes (9–11). Within frontline healthcare providers, nurses are usually the first responders to infectious disease outbreaks (8, 12). Therefore, their competencies in responding to infectious disease outbreaks are vital in preventing further transmission of infectious diseases and ensuring the safety and well-being of patients, healthcare providers, and communities as a whole.

Competency is defined as a combination of complex attributes of skills, knowledge, and attitudes, which enables individuals to perform tasks toward desired outcomes (13). To date, the competencies in responding to infectious disease outbreaks have primarily been discussed in the field of disaster nursing. In the core competencies in disaster nursing version 2.0 prospered by the International Council of Nurses (ICN), nurses are expected to possess eight competencies, including preparation and planning, communication, incident management, safety and security, assessment, intervention, recovery, and law and ethics (14). Previous studies have provided evidence suggesting that nurses’ competencies affect nursing effectiveness when responding to disaster events, regardless of the varying levels of competencies observed in these studies (15, 16). Additionally, it has been believed that adequate disaster education and training will enhance nurses’ competencies in responding to different disaster events (17). However, our understanding of the competencies in responding to infectious disease outbreaks of nurses in primary healthcare institutions is severely limited. On the one hand, most previous studies have focused on the competencies in disaster nursing for various types of hazards and have only briefly touched upon the competencies related to infectious disease outbreaks. Although these findings in disaster nursing may have some degree of relevance to infectious disease outbreaks, their applicability is generally limited and not clearly defined (10). On the other hand, while several previous studies have examined the competencies in responding to infectious disease outbreaks among nurses (18, 19), they have not specifically demonstrated the competencies required by nurses in primary healthcare institutions. Additionally, few studies have reported the specific training needs required by nurses to improve the competencies when responding to infectious disease outbreaks. This is a significant gap in the literature because primary healthcare institutions play a crucial role in providing initial and fundamental healthcare services during outbreaks of infectious diseases, especially in resource-limited areas of developing countries.

Therefore, in this study, we aimed to evaluate the competencies in responding to infectious disease outbreaks among nurses in primary healthcare institutions, as well as to identify their training needs. The findings obtained from this study will provide valuable insights to healthcare institutions and policymakers, aiding them in improving the competencies in responding to infectious disease outbreaks among nurses in primary healthcare institutions.

## 2 Materials and methods

### 2.1 Study design

This was a quantitative, cross-sectional multicentre study.

### 2.2 Settings and sample

Participants were recruited using a multi-stage stratified cluster sampling method from June to September 2022 in Sichuan Province, western China. In the first stage, we divided Sichuan Province into five regions (the Chengdu Plain, Northeast, South, and Northwest region) according to the economic and geographic status, then, calculated the ratio of registered nurses in primary healthcare institutions located in these regions. In the second stage, the Probability Proportionate to Size Sampling (PPS) method was proportional to the size of nurses in primary healthcare institutions, which was used to randomly select nine cities from the five regions. In the third stage, the cluster sampling method of PPS was used to randomly select primary healthcare institutions from each city. The exclusion criteria were as follows: (1) engaging in advanced studies in any higher authority institutions during the investigation; and (2) taking maternity leave. According to the latest data from the Health Commission of Sichuan Province, the total number of registered nurses in primary healthcare institutions in Sichuan Province is approximately 74,849 (20).

The sample size was calculated using the following formula: $n={\left(\frac{Z_{1-\alpha /2}\times \sigma }{\delta }\right)}^{2}$ , where $Z_{1-\alpha /2}=1.96$ . Song et al. (21) previously reported that the competences in responding to infectious disease of nurses in tertiary hospitals were 128.05 ± 22.23. Thus, in this study, we set *σ* = 22.23, *δ* = 2, and *DEFF* was set as 2 due to the cluster sampling process. Based on these parameters, the initial sample size was determined to be 950. Considering an 80% response rate, the final sample size was adjusted to 1,188.

### 2.3 Measurement

The competencies in responding to infectious disease outbreaks were assessed using a modified Emergency Response Competency Scale for Infectious Diseases (ERCS-ID). The ERCS-ID was originally developed by Liu et al. (22) based on the emergency response competency framework for infectious diseases introduced by Kan et al. (23). The original ERCS-ID includes three competencies: prevention, preparedness, and rescue competency. The preparedness competency includes two abilities: emergency planning and legislation. The rescue competency consists of six abilities: monitoring, reporting, medical response, public health response, risk communication, and response to specific circumstances. This original ERCS-ID totally comprises 36 items, with each item scored on a five-point Likert scale (1 represents “totally unknown” and 5 represents “very familiar”). The original ERCS-ID has good content validity and internal consistency with a content validity index of 0.870 and a Cronbach’ *α* coefficient of 0.957. For this study, we removed the item on “Precautions for participating in international emergency rescues” as it was deemed impractical for nurses in primary healthcare institutions. The modified ERCS-ID consists of 35 items (see Supplementary Table S1). According to the criteria proposed by Liu et al. (22), scores below 60% are categorized as non-performance, scores between 60 and 79% indicate moderate performance, scores of 80% or higher denote proficient ranking, and scores of 90% or higher are considered at a distinguished level. In our study, the modified ERCS-ID demonstrated good internal consistency, with a Cronbach’s *α* coefficient of 0.978.

The training needs of competencies in responding to infectious disease outbreaks were also evaluated using the same items of the modified ERCS-ID mentioned above using a five-point Likert scale. A score of “1” indicated “no need” and a score of “5” indicated “urgent need.”

### 2.4 Data collection

Sociodemographic characteristics, including age, gender, marital status, educational years, professional title, monthly income, region, employment type, night work shift, and seniority were collected. Data on individuals’ experience in infectious disease outbreak trainings were also collected. All data were collected via an online survey platform.^1^ The online survey consisted of two parts: baseline characteristics and the modified scale on competencies in responding to infectious disease outbreaks, as well as training needs for these competencies. The survey commenced with a concise introduction outlining the purpose of the study, and participants were subsequently presented with an electronic written informed consent to review and sign. Upon completion of the consent process, participants were granted access to the survey. To maintain data accuracy, logistic check rules were implemented for each item, and an integrity check was carried out for the entire survey on the online platform.

### 2.5 Data analysis

Continuous variables were described using the mean ± standard deviation (SD) or median with interquartile range (IQR) depending on their distribution. Categorical variables were described using frequency and percentage. The Mann–Whitney U test and Kruskal–Wallis H test were used to compare the differences in the competencies in responding to infectious disease outbreaks and their training needs. Multiple linear regression analysis was conducted to explore the determinants of the competencies in responding to infectious disease outbreaks. The significance level was set at a two-tailed *p* value of 0.05. All statistical analyses were performed using SPSS v24.0 (Armonk, NY, USA: IBM Corp).

### 2.6 Ethics approval statement

This study adhered to the Declaration of Helsinki. Written informed consent was delivered to the participants online before investigation. All data were kept anonymous and used in this study only. This study was approved by the Ethical Committee of West China Hospital, Sichuan University (IRB:2020-1256).

## 3 Results

### 3.1 Baseline characteristics of the participants

Table 1 presents the baseline characteristics of the participants included in this study. A total of 1,439 nurses working in 44 primary healthcare institutions participated, with a median age of 29.0. The majority of participants were female (97.4%). Most participants were married (68.0%), held a primary professional title (77.2%), and had 10 years or more of working experience in current institutions (44.6%).

**Table 1:** Characteristics of nurses in primary healthcare institutions (*N* = 1,439).

Characteristics	*N* (%)/Median (IQR)
Gender (female)	1,402 (97.4)
Age	29.0 (25.0, 35.0)
Marital status (married)	978 (68.0)
Education years	
≤12	117 (8.1)
12–16	1,320 (91.7)
>16	2 (0.1)
Ethnic minority (yes)	169 (11.7)
Professional title	
Primary	1,111 (77.2)
Intermediate	249 (17.3)
Senior	79 (5.5)
Monthly income (RMB)	
<3,000	426 (29.6)
3,000–5,000	792 (55.0)
5,000–8,000	205 (14.2)
>8,000	16 (1.1)
Region	
Chengdu plain	624 (43.4)
Northeast	329 (22.9)
South	208 (14.5)
Northwest	251 (17.4)
West	27 (1.9)
Employment type	
Contract labor	1,018 (70.7%)
Budgeted post	421 (29.3%)
Night shift (yes)	767 (53.3%)
Seniority	
<5 years	314 (21.8)
5–10 years	483 (33.6)
≥10 years	642 (44.6)
Experience in higher authority hospitals (yes)	740 (51.4%)
Experience in infectious disease emergency rescues (yes)	61 (4.2%)
Experience in other emergency rescues (yes)	88 (6.1%)
Number of emergency rescue training attended in the last 5 years	1.0 (0, 3.0)
Number of infectious disease emergency rescue training attended in the last 5 years	2.0 (0, 5.0)

### 3.2 Competencies in responding to infectious disease outbreaks and training needs

**Figure 1:**
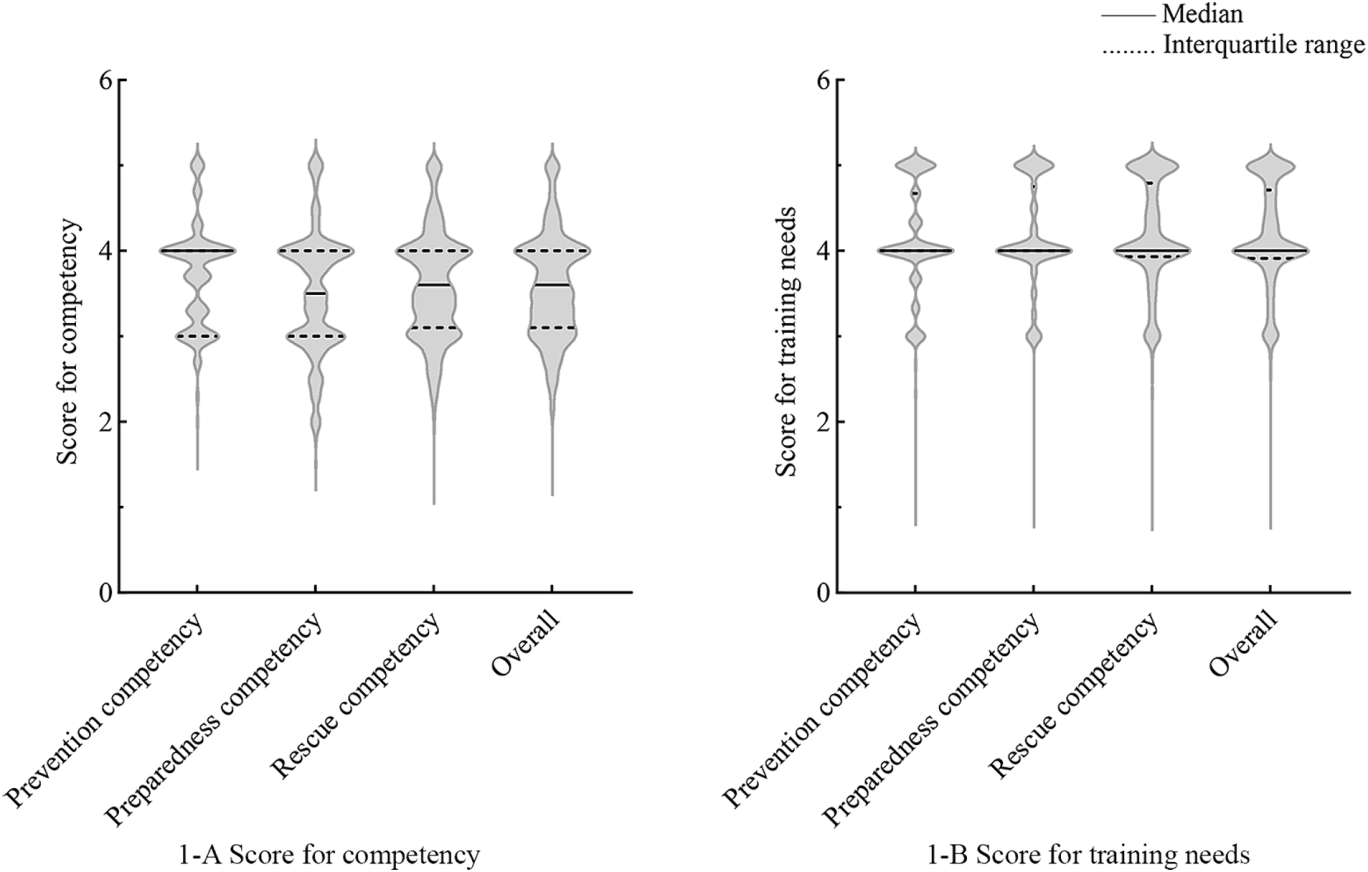
Competency and training needs of nurses in primary healthcare institutions. **(1-A)** Score for competency of nurses in primary healthcare institutions; **(1-B)** Score for training needs of nurses in primary healthcare institutions.

Table 2 shows the competencies in responding to infectious disease outbreaks, as well as the training needs of the participants. The overall competency had a median of 3.6, indicating a moderate level of performance (72.0%). The competencies were ranked based on their scores as follows: prevention competency (median = 4.0, IQR [3.0, 4.0]), rescue competency (median = 3.6, IQR [3.1, 4.0]), and preparedness competency (median = 3.5, IQR [3.0, 4.0]) (Figure 1A). In term of abilities, response to specific circumstances (median = 2.5, IQR [3.0, 4.0]) and risk communication (median = 3.0, IQR [3.0, 4.0]) attained the lowest median scores. The median of overall training needs for the competencies was 4.0 (IQR [3.9, 4.7]) (Figure 1B). The training needs for each competency and ability were at a high level, with a median score of 4.0.

**Table 2:** Competencies in responding to infectious disease outbreaks and training needs.

Dimensions	Competencies	Training needs
***Prevention competency***	4.0 (3.0, 4.0)	4.0 (4.0, 4.7)
***Preparedness competency***	3.5 (3.0, 4.0)	4.0 (4.0, 4.8)
Emergency plan	4.0 (3.0, 4.0)	4.0 (4.0, 5.0)
Legislation	3.5 (3.0, 4.0)	4.0 (4.0, 5.0)
***Rescue competency***	3.6 (3.1, 4.0)	4.0 (3.9, 4.8)
Monitor	3.3 (3.0, 4.0)	4.0 (4.0, 4.7)
Report	3.3 (3.0, 4.0)	4.0 (4.0, 4.8)
Medical response	3.5 (3.0, 4.0)	4.0 (4.0, 4.8)
Public health response	3.9 (3.4, 4.1)	4.0 (3.9, 4.9)
Risk communication	3.0 (3.0, 4.0)	4.0 (4.0, 5.0)
Response to specific circumstances	2.5 (3.0, 4.0)	4.0 (4.0, 5.0)
***Overall***	3.6 (3.1, 4.0)	4.0 (3.9, 4.7)

### 3.3 Relationship between competencies and training needs with sociodemographic characteristics

Table 3 presents the competencies in responding to infectious disease outbreaks and training needs stratified by participants’ characteristics. Participants who had experience working in higher authority hospitals scored higher competencies significantly (median = 3.6, IQR [3.2, 4.0] vs. median = 3.5, IQR [3.1, 4.0], *p* = 0.002). There were also statistically significant differences in competency scores among the participants from different regions (*p* < 0.001). In terms of training needs, no statistically significant differences were found among the participants when stratified by their general characteristics.

**Table 3:** Competencies in responding to infectious disease outbreaks and training needs stratified by general characteristics.

Variables		Frequency	Competencies	Statistics	*p* valve	Training needs	Statistics	*p* value
Gender	Male	37	3.7 ± 0.6	−0.598^†^	0.55	4.0 (3.7, 4.8)	−0.587^†^	0.626
	Female	1,402	3.6 (3.1, 4.0)			4.0 (3.9, 4.7)		
Age (years)	<30	748	3.6 (3.1, 4.0)	5.147^‡^	0.161	4.0 (3.9, 4.8)	4.163^‡^	0.244
	30–39	474	3.5 (3.1, 4.0)			4.0 (3.9, 4.7)		
	40–49	164	3.5 (3.1, 4.0)			4.0 (3.8, 4.5)		
	≥50	53	3.6 ± 0.5			4.1 (3.8, 4.8)		
Marital statues	Single	461	3.6 (3.1, 4.0)	−1.319^†^	0.187	4.0 (3.9, 4.8)	−1.374^†^	0.169
	Married	978	3.5 (3.1, 4.0)			4.0 (3.9, 4.7)		
Education years	≤12	117	3.7 (3.1, 4.0)	0.924^‡^	0.63	4.0 (4.0, 4.8)	2.021^‡^	0.364
	12–16	1,320	3.5 (3.1, 4.0)			4.0 (3.9, 4.7)		
	>16	2	3.5 (3.0, 3.5)			4.0 (4.0, 4.0)		
Professional title	Primary	1,111	3.6 (3.1, 4.0)	0.603^‡^	0.74	4.0 (3.9, 4.7)	2.587^‡^	0.274
	Intermediate	249	3.5 (3.2, 4.0)			4.0 (4.0, 4.7)		
	Senior	79	3.6 ± 0.5			4.0 (3.6, 4.6)		
Region	Chengdu plain	624	3.5 (3.1, 4.0)	21.502^‡^	<0.001	4.0 (3.9, 4.7)	6.930^‡^	0.14
	Northeast	329	3.5 (3.0, 4.0)			4.0 (3.9, 4.7)		
	South	208	3.7 (3.3, 4.0)			4.1 (3.9, 4.9)		
	Northwest	251	3.7 (3.3, 4.0)			4.0 (3.9, 4.8)		
	West	27	3.6 ± 0.2			3.8 ± 0.7		
Employment type	Contract labor	1,018	3.6 (3.1, 4.0)	2.194^†^	0.139	4.0 (3.9, 4.7)	0.595^†^	0.441
	Budgeted post	421	3.5 (3.1, 4.0)			4.0 (3.9, 4.7)		
Night shift	Yes	767	3.6 (3.1, 4.0)	−1.420^†^	0.156	4.0 (3.9, 4.7)	−0.817^†^	0.414
	No	672	3.5 (3.1, 4.0)			4.0 (3.9, 4.7)		
Seniority	<5 years	314	3.6 (3.1, 4.0)	3.093^‡^	0.213	4.0 (3.9, 4.8)	0.562^‡^	0.755
	5–10 years	483	3.6 (3.1, 4.0)			4.0 (3.9, 4.8)		
	≥10 years	642	3.5 (3.1, 4.0)			4.0 (3.9, 4.7)		
Experience working in higher authority hospitals	Yes	699	3.6 (3.2, 4.0)	−3.038^†^	0.002	4.0 (3.9, 4.7)	−0.244^†^	0.807
	No	740	3.5 (3.1, 4.0)			4.0 (3.9, 4.7)		

^†^Mann–Whitney U test; ^‡^Kruskal–Wallis H test.

### 3.4 Determinants of competencies in responding to infectious disease outbreaks

Multiple linear regression analyses revealed that age had a negative impact on the competencies in responding to infectious disease outbreaks (*β* = −0.074, *p* = 0.005). Experience working in higher authority hospitals, on the other hand, had a positive effect on the competencies (*β* = 0.057, *p* = 0.035). In addition, the location of primary healthcare institutions and number of infectious disease outbreak trainings attended within the last 5 years were also found to be significant determinants of the competencies (*β* = 0.212, *p* < 0.001) (Table 4).

**Table 4:** Determinants of competencies in responding to infectious disease outbreaks.

Variables	Std. *β*	*p* value	Adj. *R*^2^
Age	−0.074	0.005	0.064
Experience in higher authority hospitals (yes)	0.057	0.035	
Region			
Chengdu plain	Ref	–	
Northeast	−0.003	0.916	
South	0.064	0.024	
Northwest	0.092	0.001	
West	0.002	0.955	
Infectious disease outbreak trainings attended in the last 5 years	0.212	<0.001	

## 4 Discussion

In this study, we found that nurses in primary healthcare institutions possessed a moderate level of the competencies in responding to infectious disease outbreaks. Our findings indicated that these competencies were influenced by the nurses’ geographical regions, as well as their experience working in higher authority hospitals, and the infectious disease outbreak trainings they had attended within the past 5 years. Moreover, this study had identified the pressing needs for further training to enhance the competencies in responding to infectious disease outbreaks.

In this study, we found that the competencies in responding to infectious disease outbreaks were at a moderate level. Compared to the investigation conducted in 2017 (22), there is a slight increase (7.5%) in the competencies in responding to infectious disease outbreaks. Previous literature has extensively documented the positive impact of experience and training related to disasters on nurses’ competencies (17, 24, 25). Since the outbreaks of COVID-19, nurses working in primary healthcare institutions have received training in COVID-19 prevention and control, and have provided nursing care for patients with COVID-19 in clinical practice (26, 27). Their experience with the COVID-19 pandemic may help improve the competencies in responding to infectious disease outbreaks. However, within the dimension of rescue competency, abilities of risk communication and response to specific circumstances stayed at a relative low level. The findings indicate that nurses working in primary healthcare institutions struggle to effectively communicate important information about infectious disease outbreaks to the public. Additionally, they face challenges in responding to specific circumstances, such as pestilence caused by natural disasters, except for COVID-19. These two ability sets are notably below the benchmarks set by previous studies conducted by Li et al. (18) and Karnjuš et al. (28), who reported that nurses achieved scores surpassing 70% in the abilities of risk communication and response to specific circumstances. The disparity in these results could potentially be attributed to the varying proportions of nurses from tertiary hospitals and educational backgrounds. Therefore, it is imperative to improve the risk communication abilities of nurses working in primary healthcare institutions, enabling them to contribute effectively to the prevention and control of infectious diseases by disseminating key information to the public. Furthermore, this finding underscores the urgency to enhance the nurses’ ability in these institutions to respond promptly and effectively to specific circumstances arising from infectious disease outbreaks. To address this issue, simulation-based learning, which is widely utilized in nursing education (29), can be beneficial. This approach enhances the competencies by simulating specific scenarios of infectious disease outbreaks using virtual reality (VR) or augmented reality (AR). It would be particularly advantageous for nurses who have limited experience in handling infectious disease outbreaks. Moreover, this study found that the training needs for the competencies in responding to infectious disease outbreaks were at a high level, suggesting a long-term impact of the COVID-19 pandemic on nurses’ sense of crisis in primary healthcare institutions.

In our study, we discovered that nurses from the South and Northwest regions demonstrated better competencies in responding to infectious disease outbreaks. These regions in Sichuan Province are susceptible to natural disasters like earthquakes, debris flows, and landslides (30). Therefore, nurses employed in primary healthcare institutions in these areas may have more experience in disaster nursing, which could explain their enhanced competencies in responding to infectious disease outbreaks, as one aspect of disaster nursing. In this study, we also found that nurses who had experience in higher authority hospitals demonstrated better competencies in responding to infectious disease outbreaks. These results confirm previous findings (17, 24, 25) and suggest the importance of training to improve nurses’ competencies in responding to infectious disease outbreaks. In China, training for nurses in primary healthcare institutions is particularly important due to the relatively weak quality of medical human resources in these settings. For example, our study found that only 0.1% of nurses hold a master’s degree and 5.5% have a senior professional title, which is lower than national average level (31). No statistically significant differences were observed in the training needs, indicating the training for competencies in responding to infectious disease outbreaks reaches a consensus in all participants.

This study found that the competencies in responding to infectious outbreaks were influenced by several factors. Nurses’ experience in higher authority hospitals and infectious disease outbreak trainings attended had positively impacts on the competencies in responding to infectious disease outbreaks. These findings are consistent with previous studies (17, 24). However, this study found that age was negatively associated with the competencies in responding to infectious disease outbreaks. In China, higher nursing education has gradually developed in the last two decades, resulting in younger nurses usually having longer education years and less clinical experience. This may explain the negative association between educational years and the competencies in responding to infectious disease outbreaks. However, the age of nurses is not strictly associated with the number of educational years due to the availability of continuous education programs. Further research is required to clarify the associations between age and the competencies in responding to infectious disease outbreaks. Furthermore, the present study revealed that the South and Northwest regions exhibit favorable effects on the competencies in responding to infectious disease outbreaks, likely due to the nurses’ extensive experience in disaster nursing within these regions. Consequently, it is imperative for managers and policy makers to acknowledge this regional disparity in competencies pertaining to infectious disease response. They should prioritize the implementation of evidence-based, competency-driven, and high-quality training programs for nurses employed in primary healthcare institutions situated in regions with lower competencies, with the aim of enhancing their competencies to a comparable level (32, 33).

This study has some limitations. First, due to feasibility concerns, the sample only included nurses from primary healthcare institutions in cities and towns. This may limit the findings’ generalizability. Second, because this online survey does not allow for face-to-face communication to explain the meaning of each item, the content may be misunderstood. Third, the participants self-assessed their competencies so we have no way of knowing to what extent their self-assessment matched with actual competencies. In addition, this study was conducted following the COVID-19 pandemic. Due to the lack of prior knowledge about the virus, nurses had to rapidly learn and enhance their skills to respond to this novel infectious disease. In this process, nurses’ competencies changed over time and may have been influenced by various factors, such as the quality of training, role assignments, and local conditions, which may introduce bias. Despite these limitations, the study found moderate competencies in responding to infectious disease outbreaks and identified factors that influence these competencies, and demonstrated the high training needs for the competencies in responding to infectious disease outbreaks in nurses working in primary healthcare institutions. In the future, studies adopting an explanatory mixed methods approach may be beneficial for further exploration of the experiences, competencies, and training needs of nurses in primary healthcare institutions when responding to infectious disease outbreaks.

## 5 Conclusion

This study discovered that the competencies of nurses in primary healthcare institutions in responding to infectious disease outbreaks were at a moderate level, which were influenced by varied factors. To enhance their competencies, professions could strengthen nursing education tailored specifically to infectious disease response and implementing simulation-based training. Furthermore, it is of paramount importance to consider regional differences in nurses’ competencies when allocating educational and training resources in order to achieve overall improvement in the competencies of nurses across all primary healthcare institutions.

## Data availability statement

The raw data supporting the conclusions of this article will be made available by the authors, without undue reservation.

## Ethics statement

The studies involving humans were approved by the Ethical Committee of West China Hospital, Sichuan University. The studies were conducted in accordance with the local legislation and institutional requirements. The participants provided their written informed consent to participate in this study.

## Author contributions

WZ: Conceptualization, Data curation, Formal analysis, Methodology, Writing – original draft, Writing – review & editing. JZ: Data curation, Formal analysis, Investigation, Writing – original draft, Writing – review & editing. LY: Data curation, Formal analysis, Investigation, Writing – original draft, Writing – review & editing. JL: Conceptualization, Formal analysis, Methodology, Resources, Supervision, Writing – review & editing. HG: Conceptualization, Formal analysis, Funding acquisition, Investigation, Methodology, Resources, Supervision, Writing – original draft, Writing – review & editing.

## Footnotes

^1^ www.wjx.cn
