# Supplementary material for: Competency in responding to infectious disease outbreaks among nurses in primary healthcare institutions: a quantitative, cross-sectional multicentre study
Source: Front Public Health. 2024 Jul 22;12:1406400. doi: 10.3389/fpubh.2024.1406400 (PMC11298484; doi:10.3389/fpubh.2024.1406400)
Supplement: Supplementary file 1 [file Table_1.DOCX]

Supplementary Material

# Supplementary Table S1. The Modified Emergency Response Competency Scale for Infectious Diseases (ERCS-ID)

# [*Note*] Please type "√" on the corresponding number according to the actual familiarity of the content listed in the table. Each item is scored on a five-point Likert scale (1 represents "totally unknown" and 5 represents "very familiar").

| No. | Item | Item content | 1 | 2 | 3 | 4 | 5 |
| --- | --- | --- | --- | --- | --- | --- | --- |
| 1 | A1 | The infectious disease spectrum (the concept and significance of incubation period, infectious period, recessive infection, dominant infection, etc.) |  |  |  |  |  |
| 2 | A2 | The spread of infectious diseases. |  |  |  |  |  |
| 3 | A3 | The principles of prevention and control of infectious diseases. |  |  |  |  |  |
| 4 | B1 | The responsibilities of health workers in the *National Emergency Plan for Public Health Emergencies* |  |  |  |  |  |
| 5 | B2 | The responsibilities of health workers in the *National Emergency Plan for Medical and Health Rescue in Public Emergencies.* |  |  |  |  |  |
| 6 | B3 | The *Regulations on Public Health Emergencies.* |  |  |  |  |  |
| 7 | B4 | The *Law of the People's Republic of China on the Prevention and Control of Infectious Diseases* |  |  |  |  |  |
| 8 | C1 | The significance of symptom monitoring. |  |  |  |  |  |
| 9 | C2 | The definition of syndrome and target disease |  |  |  |  |  |
| 10 | C3 | Classifying patients according to established case definitions in emergencies. |  |  |  |  |  |
| 11 | C4 | The time limit for reporting legal infectious diseases. |  |  |  |  |  |
| 12 | C5 | How to fill in the Report Card of Infectious Diseases of the People's Republic of China correctly. |  |  |  |  |  |
| 13 | C6 | The scope of information regarding the reporting of infectious disease emergencies. |  |  |  |  |  |
| 14 | C7 | The reporting process of public health emergencies. |  |  |  |  |  |
| 15 | C8 | The basic knowledge of medical response to infectious disease emergencies. |  |  |  |  |  |
| 16 | C9 | Implementing patient specimen collection regarding infectious diseases correctly. |  |  |  |  |  |
| 17 | C10 | The precautions for specimen preservation and transportation. |  |  |  |  |  |
| 18 | C11 | Having clear and reliable sources of information regarding infectious diseases. |  |  |  |  |  |
| 19 | C12 | How to obtain key information from selected information sources |  |  |  |  |  |
| 20 | C13 | The methods of psychological self-adjustment after caring for patients with infectious diseases. |  |  |  |  |  |
| 21 | C14 | The meaning of standard precautions |  |  |  |  |  |
| 22 | C15 | The protection requirements of various transmission routes of infectious diseases. |  |  |  |  |  |
| 23 | C16 | Putting on and taking off personal protective equipment correctly. |  |  |  |  |  |
| 24 | C17 | The emergency treatment methods for exposure to the patient's blood and body fluids. |  |  |  |  |  |
| 25 | C18 | The principles of setting up infectious disease wards. |  |  |  |  |  |
| 26 | C19 | Properly implementing hand hygiene. |  |  |  |  |  |
| 27 | C20 | Properly disposing medical waste related to infectious diseases. |  |  |  |  |  |
| 28 | C21 | Properly disposing death bodies of potential and actual infectious disease patients. |  |  |  |  |  |
| 29 | C22 | The method of environmental disinfection. |  |  |  |  |  |
| 30 | C23 | The isolation principles of various transmission channels. |  |  |  |  |  |
| 31 | C24 | Properly implementing the isolation of patients with various infectious diseases. |  |  |  |  |  |
| 32 | C25 | The quarantine methods for close contacts of various infectious diseases |  |  |  |  |  |
| 33 | C26 | Formulating health education programs to control the spread of infectious diseases. |  |  |  |  |  |
| 34 | C27 | The appropriate response to a bioterrorism attack. |  |  |  |  |  |
| 35 | C28 | The emergency response to infectious diseases after natural disasters. |  |  |  |  |  |
